# Single blind, randomized study comparing clinical equivalence of Trusilk ^®^ and Mersilk ^®^ silk sutures for mucosal closure following surgical removal of mesioangular impacted mandibular third molar

**DOI:** 10.12688/f1000research.122678.1

**Published:** 2022-06-21

**Authors:** Ramdas Balakrishna, Dharnappa Poojary, Arvind R, Shrikanth Sali, Ashok Kumar Moharana, Deepak TS

**Affiliations:** 1 Department of Oral & Maxillofacial Surgery, KLE Society’s Institute of Dental Sciences & Research Center, Bangalore, Karnataka, 560022, India; 2 Department of Oral & Maxillofacial Surgery, Manipal College of Dental Sciences Mangalore, Manipal Academy of Higher Education(MAHE), Mangalore, Karnataka, 575001, India; 3 Clinical Affairs, Healthium Medtech Limited, Bangalore, Karnataka, 560058, India

**Keywords:** Impacted third molar, Mucosal suturing, Silk suture, Pain, Swelling, Trismus

## Abstract

**Background:** Mesioangular impacted mandibular third molar is a common dental anomaly, for which surgical extraction is required. Post-surgery closure of mucosa reduces the prevalence of pain and other surgery-associated complications. We compared tissue reaction/inflammation after 3 and 7 days of mucosal closure with Trusilk ^®^ and Mersilk ^®^ silk sutures, following impacted mandibular third molar removal.

**Methods: **This multicenter, prospective, two-arm, parallel-group, randomized (1:1), single-blind study (July 2020-November 2021) included subjects (Trusilk ^®^, n=65 and Mersilk ^®^, n=64), requiring mucosal suturing following impacted mandibular third molar removal. The primary endpoint, incidence of pain, swelling and trismus at the extraction area on post-surgery day 3 and 7 was evaluated. The secondary endpoints, incidence of tissue reaction, wound infection, suture loosening, other complications, operative time, amount of anesthesia, intraoperative suture handling, time needed for complete wound healing and suture removal, and adverse events were also recorded.

**Results: **Socio-demographic and intra-oral characteristics were comparable between the groups. In Trusilk ^®^ and Mersilk ^®^ groups, a gradually decreasing pain score, starting from day 0 post-surgery (42.17±22.38 vs. 45.97±22.20) to day 7 (8.40±11.93 vs. 8.28±12.13) to day 30 (1.98±0.89 vs. 1.75±0.76) was witnessed. After the surgery, 21.54% and 17.19% subjects in Trusilk ^®^ and Mersilk ^® ^groups, respectively, had no post-operative swelling, while at the last two visits none of the subjects had swelling. Non-significant difference in wound infection, suture loosening, wound healing, bleeding, taste changes, operative time, amount of anesthesia, intraoperative suture handling, and time needed for complete wound healing and suture removal was noted among the groups. No suture-related adverse events were recorded.

**Conclusions: **The results indicated that the Trusilk ^®^ and Mersilk ^®^ silk sutures are clinically equivalent and can be used for mucosal closure after removal of an impacted mandibular third molar with a minimal rate of pain, swelling and trismus.

**Clinical Trial Registry of India Registration:** CTRI/2020/03/024100 (20/03/2020)

## Introduction

Impacted tooth is a common dental anomaly that usually occurs in the mandibular third molars, maxillary canines, and maxillary second premolars. ^1^ Mandibular third molar is commonly impacted (72%), in comparison to maxillary third molars (37.9%), reason for which can either be the lack of space and physical barrier posed by the permanent second molar, or malalignment due to delayed mineralization and early physical maturation. ^2^ In addition, genetic characteristics and food habits also contribute to the occurrence of tooth impaction. ^3^ The prevalence of third molar impaction has already been reported in various countries, such as India, Pakistan, Iran, Saudi Arabia, Oman, Turkey and Sweden. ^4^

Dental surgeons prefer third molar extraction based on pain, recurrent infection, local and/or regional swelling, dental caries or cysts, etc. ^5^^,^^6^ In the oral surgical field, surgical removal of third molar is considered as a common dental operative procedure, ^4^ which involves accessibility of the tooth by removing the overlying bone and exposing the tooth. After sectioning, the sectioned tooth is delivered following closure of the wound. ^7^ The associated complications of impacted tooth surgery are pain, paresthesia (i.e., damage to sensory nerve), dry socket, infection, and hemorrhage. Several factors such as patient’s age, health status, and position of the tooth further influence the rate of complications. ^8^

Following the surgery, primary tissue closure or mucosal closure using a braided silk suture has been found to reduce pain, edema, and trismus, and improve wound healing. ^9^ In comparison to other non-absorbable suture materials, silk suture is mostly used for dental procedures due to its cost-effectiveness. ^10^ The present study was designed to evaluate clinical equivalence of two common silk suture brands, Trusilk ^®^ and Mersilk ^®^ for mucosal closure in subjects undergoing planned surgical removal of mesioangular impacted mandibular third molar.

## Methods

### Study design

This was a multicentric, prospective, two-arm, parallel-group, randomized (1:1), single-blind study, conducted in two different centers between July 2020 and November 2021. The primary objective of the study was to compare tissue reaction/inflammation with Trusilk ^®^ and Mersilk ^®^ silk sutures post primary closure of mucosa in subjects undergoing planned surgical removal of impacted mandibular third molar at 3 and 7 days. The secondary objectives were to compare the incidence of tissue reaction and infection, the effect of suture material on wound healing and other surgical outcomes, overall intraoperative handling, and the common post-surgical complications between the two groups, Trusilk ^®^ and Mersilk ^®^.

### Ethical approval

This clinical study was registered on 20 ^th^ March 2020 with Clinical Trial Registry of India (CTRI Registration No- CTRI/2020/03/024100). The institutional ethics committee of both participating sites approved this study protocol (K.L.E Society’s Institute of Dental Sciences Ethics Committee approved the study on 21st November 2019 with approval number KIDS/IEC/NOV-19/3; and Manipal Academy of Higher Education Ethics Committee approved the study on 8th February 2020). The study was designed, conducted, recorded, and reported in compliance with the principles of International Conference on Harmonization of Technical Requirements-Good Clinical Practice (ICH-GCP E6 R2) guidelines, EN ISO 14155:2020 guidelines, Indian MDR rules 2017, MDR (EU) 2017/745, Indian New Drugs and CT rules 2019, and Consolidated Standards of Reporting Trials (CONSORT). ^19^

### Informed consent

Written informed consent was obtained from all participants for participation in the study as well as for publication of their clinical data.

### Study participants

Male and female subjects, aged 20-40 years with mesioangular impacted mandibular third molar, requiring mucosal suturing after removal, who visited Department of Oral & Maxillofacial Surgery of both the centers were invited to participate in this research. They were included after obtaining informed consent. Subjects only with American Society of Anesthesiology classification of grade I or II were included.

Subjects with infected molar or complicated impacted third molar, pregnant or lactating women and subjects, who were unlikely to comply with surgical procedure or complete the scheduled visits in the opinion of the Investigators were excluded. Subjects with a history of allergy to silk or similar products, and a history of systemic diseases (diabetes mellitus, tuberculosis, bleeding disorders, osteoporosis, unstable or life-threatening conditions), or undergoing radiation therapies were also excluded. Subjects receiving any type of local and systemic drugs, or drugs like aspirin, blood thinners and anticoagulant therapy, or an experimental drug or used an experimental medical device within 30 days prior to surgery, or who had the habit of drug abuse were excluded. Subjects who were already participating in another trial or had direct involvement in the proposed study or other studies under the direction of that Investigator or study center were excluded.

### Study settings

The study was conducted at two sites: (i) The Department of Oral & Maxillofacial Surgery, KLE Society’s Institute of Dental Sciences & Research Center, Bangalore, India, and (ii) The Department of Oral & Maxillofacial Surgery, Manipal College of Dental Sciences, Mangalore, India.

### Intervention

Both Trusilk ^®^ (Healthium Medtech Limited) and Mersilk ^®^ (Ethicon, Johnson & Johnson) are natural non-absorbable black braided sterile silk suture. Both sutures are indicated for use in soft tissue approximation and or ligation. Both the sutures are composed of an organic protein called fibroin (derived from the domesticated species *Bombyx mori*) and are coated with wax to reduce friction.

### Study outcomes

***Demographics and other relevant characteristics***

Age, gender, ethnicity, weight, height, body mass index, history of smoking, gutkha chewing (tobacco use), education and employment status were recorded at screening visit. The vital signs like pulse rate, respiration rate, and systolic and diastolic blood pressure (measured after the subjects were in supine position for 5 minutes) were recorded. A general medical or surgical history was also noted. Intraoral examination for present teeth, missing teeth, interincisal mouth opening, tongue size (macroglossia/normal/microglossia), dental scaling, dental caries, tooth deposits, and oral/dental cyst was done. Diagnosis of impacted third molar was done by dental X-rays.

***Primary endpoints***

The primary endpoint was incidence of clinical inflammation/tissue reaction in the extraction area at 72 hours (3 days) and 7 days post-surgery. It was identified with assessment of pain, swelling and trismus. Visual analogue scale (VAS) score for pain was recorded at screening, post-surgery (Day 0), and on all follow-up days. VAS of 0–4 was graded as no pain, 5-44 as mild pain, 45-74 as moderate pain, and 75–100 as severe pain. Swelling and trismus were also assessed using a VAS scale ranging from 0 to 5, where 0 is no swelling, 1 is slight swelling, 2 is mild swelling, 3 is severe swelling, 4 is very severe swelling, and 5 is extremely severe swelling.

***Secondary endpoints***

The secondary endpoints, included incidence of tissue reaction, wound infection, suture loosening, other complications (delayed wound healing, bleeding, and taste changes), operative time (time from oral incision to the end of wound closure), amount of anesthesia used, intraoperative suture handling, time needed for complete wound healing and suture removal. Other postoperative complications, including dry socket, paresthesia, taste changes, suture sent for culture (in case of microbial deposits on sutures), and adverse events were also assessed. Any untoward medical occurrence, unintended disease or injury, or untoward clinical signs, which were already reported as study endpoints, were not labelled and reported as adverse events. All the medications prescribed to the subjects during the study period were also registered.

The handling characteristics viz. ease of passage through tissue; first-throw knot holding; knot tie-down smoothness; knot security; surgical handling; memory; suture fraying of both sutures were rated on a five-point scale as follows: 1 poor, 2 fair, 3 good, 4 very good, and 5 excellent. Wound healing was measured by using a healing index proposed by Landry, Turnbull and Howley ^11^ to describe the extent of clinical healing after surgery. The scale evaluated tissue color, suppuration, granulation tissue, bleeding on palpation and incision margins on a five-point scale: 1 is very poor, 2 is poor, 3 is good, 4 is very good, and 5 is excellent. Bleeding assessment was done on a modified VAS scale, on which 0 corresponds to no bleeding, 1 to oozing, 2 to accidental low bleeding, 3 to continuous low bleeding and 4 to massive bleeding.

### Sample size

Assumptions for sample size calculations were based on clinical data reported by Chaudhary *et al*., 2012. ^12^ Mean VAS score for swelling on Day 1 and Day 3 was reported as 2.58 and 2.08 respectively with a SD of 0.79. Assuming type I error as 5% (α=0.05) and power as 80% (1-β=0.8) and non-inferiority margin as 10%, the minimum sample size requirement was calculated to be 50 in each arm. Mean VAS score for pain on Day 1 and Day 3 was reported as 2.25 and 1.58 respectively with a SD of 0.67. Assuming type I error as 5% and power as 80% and non-inferiority margin as 10% of the difference (δ), the minimum sample size requirement was calculated to be 20 in each arm. The sample size was found to be maximum when the Day 3 rather Day 7 VAS scores were considered vis-a-vis Day 1 scores. So, a sample size of 50 in each arm could safely be adopted to address both pain and swelling (primary endpoint). Further, considering a drop out of 20% and post-randomization exclusion of 10% the required sample size was increased to 66 in each arm. So, a total of 132 subjects participated in this trial, with 66 subjects randomized to Trusilk ^®^ arm and 66 to Mersilk ^®^ arm.

Sample size calculation formula:

$$\text{Non}-\text{inferiority}\;\mu_{1}-\mu_{2}\geq \delta \;\mu_{1}-\mu_{2}<\delta \;n_{i}=\frac{{\left(Z_{\alpha }+Z_{\beta }\right)}^{2}\;\sigma^{2}}{{\left(\mu_{1}-\mu_{2}-\delta \right)}^{2}}$$

*n*_*i*_: Sample size required in each group, *Z*_*α*_: Conventional multiplier for alpha, *Z*_*β*_: Conventional multiplier for power, *μ*_1_: Mean score of swelling and pain in the standard Mersilk ^®^ arm, *μ*_2_: Mean score of swelling and pain in Trusilk ^®^ arm, *δ*: Margin of non-inferiority difference, *σ*: Standard deviation.

### Randomization and blinding

Two random lists of the size n=66 (33 vs. 33) were generated by using version 1.0 of Random Allocation Software, using block sizes of 4, 6 or 8. Subjects were randomized using block randomization to ensure an unbiased treatment assignment in a 1:1 ratio to receive either the Trusilk ^®^ or Mersilk ^®^ suture. The randomization concealment was done by SNOSE (Sequentially Numbered Opaque Sealed Envelopes) technique and codes were issued to the sites in sealed envelopes.

This was a single-blind study and the subjects were kept blinded to the device allocation status. Due to the nature of the intervention, the operating staff could not be blinded to allocation but they were instructed not to disclose the allocation status of the participant at any time.

### Study procedure

Post-screening (screening visit), the study participants underwent surgical procedures (baseline visit, Day 0). All routine aseptic precautions according to the existing standards were followed before, during and after the surgery. The subjects were treated as per the standard of care, after transferring to the observation room. An inflammation recording card was given to subjects after surgery to manually mark the level of pain, swelling and trismus, after onset of symptoms at the home. The subjects were followed-up on Day 3 (In-person visit), Day 7 (In-person visit), Day 12 (In-person visit), Day 30 (Telephonic visit) and Month 3 post-surgery (Telephonic visit) to record the primary and secondary endpoints.

### Statistical analysis

The subjects were analyzed using the per-protocol or PP analysis set, which consist of all subjects who had complete data on the primary effectiveness parameter at 3 months of follow-up. All continuous variables were expressed as mean±SD (standard deviation). The normally distributed data were compared using t-test and distribution-free data was compared using Kruskal–Wallis test. All qualitative variables were expressed as proportions or percentages and were compared using Chi-square test or Fisher's Exact test. A p-value of < 0.05 was considered statistically significant. The primary endpoint was summarized as mean±SD (pain VAS score) and proportion/ percentage of subjects with no pain, swelling and trismus. Mean values were compared using Kruskal–Wallis test and proportion was compared using Chi-square test. The secondary endpoints were expressed as mean±SD or as proportions/percentages based on quantitative or qualitative nature of the variable. All analyses were carried out with SPSS version 28.0 (SPSS, Chicago, Illinois, USA, RRID:SCR_016479).

## Results

**Figure 1.**
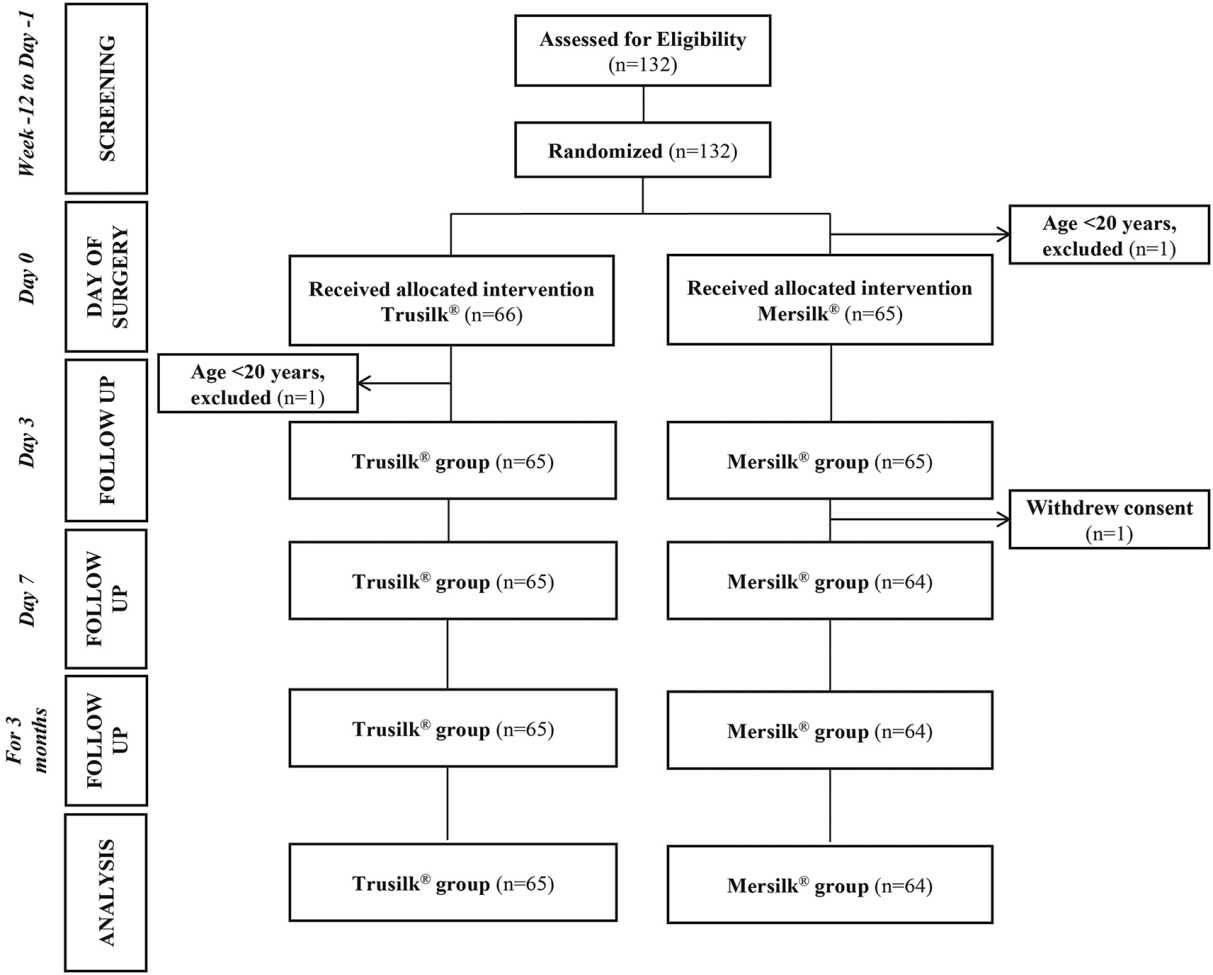
CONSORT flow chart.

Between July 2020 and August 2021, a total of 132 subjects were screened. One participant from each group was excluded and reported as a protocol violation (age <20 years). One participant from Mersilk ^®^ group was also excluded because of consent withdrawal. Data was analyzed for a total of 129 subjects (Trusilk ^®^, n=65; Mersilk ^®^, n=64), who completed the study ( Figure 1). ^18^ The follow-up of the last subject was completed in November 2021.

### Baseline demographics and other relevant characteristics

All the subjects who participated in the trial were Indian. Out of all participants, 84 (65.12%) were women and 45 (34.88%) were men ( Table 1). The education level of the participants was comparable between the groups, with 57 (87.69%) in Trusilk ^®^ group, and 59 (92.19%) subjects in Mersilk ^®^ group who attended college and above. Overall, 35 (53.85%) and 32 (50.00%) subjects in Trusilk ^®^ and Mersilk ^®^ groups, respectively, were employed. In total, four (6.15%) subjects in Trusilk ^®^ group, and one (1.56%) subject in Mersilk ^®^ group had smoking history, while one subject in each Trusilk ^®^ (1.54%) and Mersilk ^®^ (1.56%) group had a habit of gutkha chewing. Baseline demographics and vital signs were comparable between the treatment groups ( Table 1). The participants had no medical or surgical history.

**Table 1.** Baseline demographics and intra oral characteristics of the study participants.

Baseline characteristics		Trusilk ^®^ (n=65)	Mersilk ^®^ (n=64)	p value
***Demographics***				
Gender, n (%)	*Males*	22 (33.85)	23 (35.94)	0.80
	*Females*	43 (66.15)	41 (64.06)	
Age (years), Mean±SD		27.96±5.75	27.87±6.14	0.93
***Anthropometrics***				
Weight (kg), Mean±SD		62.53±11.28	61.86±12.43	0.75
Height (cm), Mean±SD		162.03±10.02	161.69±10.35	0.85
BMI (kg/m ^2^), Mean±SD		23.88±4.14	23.59±3.98	0.69
***Vital signs***				
Pulse rate (beats/minute), Mean±SD		84.08±10.12	85.52±10.32	0.43
Respiratory rate (respiration/minute), Mean±SD		15.63±1.59	15.95±1.41	0.23
Systolic blood pressure (mmHg), Mean±SD		121.69±4.57	121.48±6.04	0.83
Diastolic blood pressure (mmHg), Mean±SD		80.91±3.84	80.53±4.43	0.61
***Intra oral examination***				
Teeth present, Mean±SD		31.09±1.30	31.11±1.61	0.95
Teeth missing, Mean±SD		0.89±1.30	0.83±1.43	0.79
IMO (mm), Mean±SD		40.74±4.65	41.83±5.10	0.21
Dental caries, n (%)	*Absent*	44 (67.69)	39 (60.94)	0.42
	*Present*	21 (32.31)	25 (39.06)	
Teeth deposits, n (%)	*Absent*	50 (76.92)	57 (89.06)	0.07
	*Present*	15 (23.08)	7 (10.94)	
Dental scaling, n (%)	*No*	61 (93.85)	61 (95.31)	0.71
	*Yes*	4 (6.15)	3 (4.69)	
VAS pain score, Mean±SD		27.86±18.95	26.63±22.14	0.42
Grade of pain, n (%)	*Nil*	8 (12.31)	15 (23.44)	0.24
	*Mild*	46 (70.77)	34 (53.13)	
	*Moderate*	10 (15.39)	14 (21.88)	
	*Severe*	1 (1.54)	1 (1.56)	
Pain before surgery, n (%)	*No*	7 (10.77)	13 (20.31)	0.13
	*Yes*	58 (89.23)	51 (79.69)	

SD: Standard Deviation, BMI: Body Mass Index, IMO: Interincisal Mouth Opening, VAS: Visual Analogue Scale.

***Intra oral examination***

The tongue size of all the study participants appeared normal. Full set of adult teeth was evident in majority of subjects of both groups, followed by 31 and 30 teeth. Average number of present and missing teeth along with findings of other intra oral examination is summarized in Table 1. Oral/dental cyst was absent in all the study participants. Before the surgery, mild swelling was recorded in 1 (1.54%) subject of Trusilk ^®^ group and 3 (4.69%) subjects of Mersilk ^®^ group, while mild trismus was noted in 2 (3.08% in Trusilk ^®^ group and 3.13% in Mersilk ^®^ group) subjects of both groups. Pain in the subjects prior to surgery is presented in Table 1.

### Primary endpoint analysis

**Figure 2.**
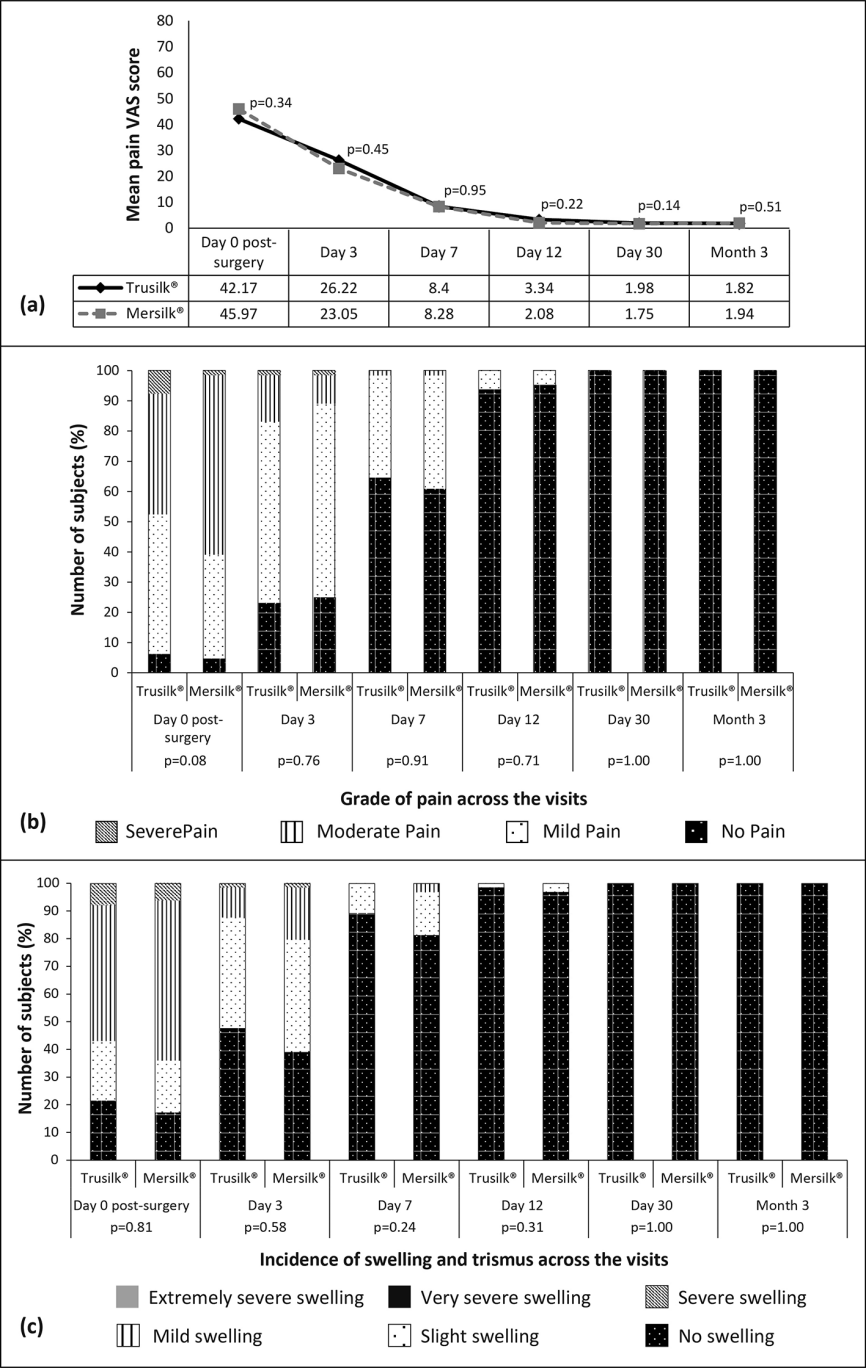
Pain (a & b), swelling and trismus (c) in Trusilk ^®^ (n=65) and Mersilk ^®^ (n=64) group. VAS: Visual Analogue Scale.

The pain was assessed using VAS scale on day 1 after recovery from anesthesia (post-surgery), on day 3 and 7, as well as on all follow-up visits. The pain score was highest on the day of the surgery (42.17±22.38 vs. 45.97±22.20) in both Trusilk ^®^ and Mersilk ^®^ group and declined steadily with each passing visit ( Figure 2a and 2b). In Trusilk ^®^ and Mersilk ^®^ groups, at day 3 and day 7 the mean pain VAS score was recorded as 26.22±20.64 vs. 23.05±19.09, and 8.40±11.93 vs. 8.28±12.13, respectively. At day 0, 3 and 7 visits, 4 (6.15%), 15 (23.08) and 42 (64.62) subjects of Trusilk ^®^ group, and 3 (4.69%), 16 (25.00) and 39 (60.94%) subjects of Mersilk ^®^ group had no pain. Similarly, the rate of swelling and trismus was also improved across the follow-up visits ( Figure 2c). At day 0 visit, the incidence of no swelling was recorded in 14 (21.54%) and 11 (17.18%) subjects of Trusilk ^®^ and Mersilk ^®^ groups, respectively. At day 3 and 7 visits, 31 (47.69%) and 58 (89.23%) subjects respectively in Trusilk ^®^ group, and 25 (39.06%) and 52 (81.25%) subjects, respectively, in Mersilk ^®^ group had no swelling. Results of pain, swelling and trismus showed non-significant differences between the groups.

### Secondary endpoint analysis

***Intraoperative profile***

**Figure 3.**
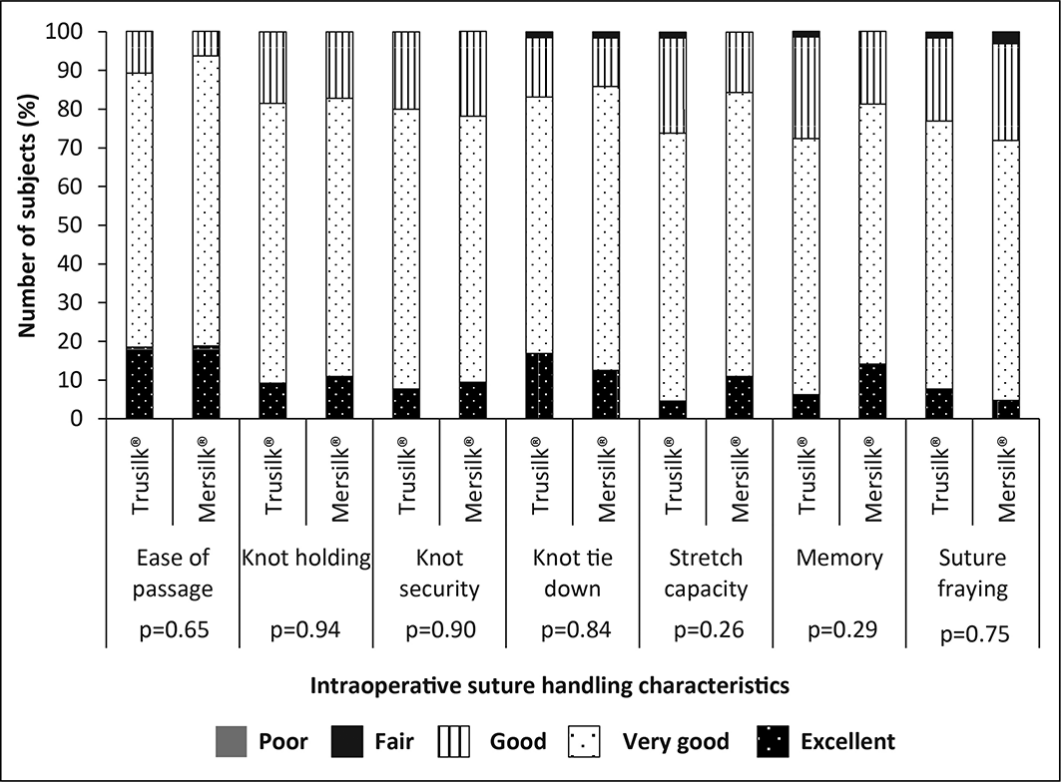
Intraoperative suture handling characteristics in study participants randomized to Trusilk ^®^ (n=65) and Mersilk ^®^ (n=64) group.

Lignocaine+Adrenaline was used for local anesthesia during the surgery in all subjects. The amount of anesthesia and active pharmaceutical ingredients strength of the anesthesia is given in Table 2. One suture was used in all subjects of the Trusilk ^®^ as well as Mersilk ^®^ group. In all subjects, 3/8 circle reverse cutting needle was used. Total operative time, and time of onset of pain, swelling and trismus after tooth extraction are comparable between the studied groups ( Table 2). The intraoperative suture handling characteristics in Trusilk ^®^ and Mersilk ^®^ groups are shown in Figure 3. The result of suture handling characteristics viz., ease of passage, knot holding, knot security, knot tie-down, stretch capacity, memory and suture fraying were marked as “excellent” or “very good” or “good” or “fair” and were comparable between the groups. None of the suture handling characteristics was graded as “poor” ( Table 3).

**Table 2.** Intraoperative and post-operative profile of the study participants.

Subject characteristics		Trusilk ^®^ (n=65)	Mersilk ^®^ (n=64)	p value
***Intraoperative***				
Amount of local anesthesia used (mL), Mean±SD		3.24±0.60	3.28±0.95	0.62
API strength of anesthesia (mg), Mean±SD		58.15±10.92	59.06±17.04	0.62
Length of operation (minutes), Mean±SD		50.83±18.66	51.53±20.63	0.79
Number of sutures used, Mean±SD		1.00±0	1.00±0	1.00
Time of onset of post-operative pain after tooth extraction (hours), Mean±SD		4.62±3.38	4.52±3.04	0.85
Time of onset of swelling and trismus after tooth extraction (hours), Mean±SD		3.99±2.97	3.59±2.67	0.40
***Post-operative***				
Number of analgesics prescribed, Mean±SD	*Day 0*	1.49±0.62	1.52±0.64	0.83
	*Day 3*	1.09±0.49	1.08±0.65	0.89
	*Day 7*	0	0.02±0.13	0.32
	*Day 12*	0.03±0.25	0.02±0.13	0.66
Wound infection, n (%)	*Day 3*	0	0	-
	*Day 7*	0	0	-
	*Day 12*	1 (1.54)	0	0.32
	*Day 30*	1 (1.54)	0	0.32
	*Month 3*	1 (1.54)	0	0.32
Suture loosening, n (%)	*Day 3*	0	1 (1.56)	0.31
	*Day 7*	4 (6.15)	3 (4.69)	0.71
	*Day 12*	0	0	-
Abnormal taste change, n (%)	*Day 3*	1 (1.54)	0	0.32
	*Day 7*	0	0	-
	*Day 12*	1 (1.54)	0	0.32
	*Day 30*	0	0	-
	*Month 3*	0	1 (1.56)	0.31
Time to complete wound healing (days), Mean±SD		5.68±3.55	6.58±4.60	0.50
Time needed for suture removal (days), Mean±SD		7.32±0.94	7.14±0.73	0.22

SD: Standard Deviation, API: Active Pharmaceutical Ingredients.

**Table 3.** Intraoperative suture handling parameters of the study participants.

Study group	Intraoperative suture handling	Ease of passage n (%)	Knot holding n (%)	Knot security n (%)	Knot tie down n (%)	Stretch capacity n (%)	Memory n (%)	Suture fraying n (%)
**Trusilk ^®^ (n = 65)**	*Excellent*	12 (18.46)	6 (9.23)	5 (7.69)	11 (16.92)	3 (4.61)	4 (6.15)	5 (7.69)
	*Very good*	46 (70.77)	47 (72.31)	47 (72.31)	43 (66.15)	45 (69.23)	43 (66.15)	45 (69.23)
	*Good*	7 (10.77)	12 (18.46)	13 (20.00)	10 (15.39)	16 (24.62)	17 (26.15)	14 (21.54)
	*Fair*	0	0	0	1 (1.54)	1 (1.54)	1 (1.54)	1 (1.54)
	*Poor*	0	0	0	0	0	0	0
**Mersilk ^®^ (n = 64)**	*Excellent*	12 (18.75)	7 (10.94)	6 (9.38)	8 (12.50)	7 (10.94)	9 (14.06)	3 (4.69)
	*Very good*	48 (75.00)	46 (71.88)	44 (68.75)	47 (73.44)	47 (73.44)	43 (67.19)	43 (67.19)
	*Good*	4 (6.25)	11 (17.18)	14 (21.88)	8 (12.50)	10 (15.63)	12 (18.75)	16 (25.00)
	*Fair*	0	0	0	1 (1.56)	0	0	2 (3.13)
	*Poor*	0	0	0	0	0	0	0
**p value**		0.65	0.94	0.90	0.84	0.26	0.29	0.75

***Post-operative profile***

**Figure 4.**
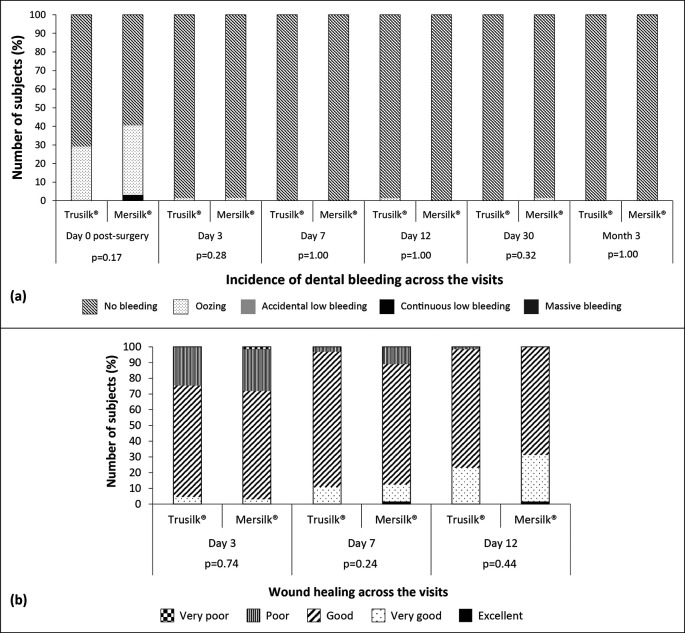
Dental bleeding (a) and wound healing (b) in Trusilk ^®^ (n=65) and Mersilk ^®^ (n=64) group.

Similar to the results of primary endpoint, pain score, swelling and trismus were improved in the next post-operative visits (day 12, day 30 and month 3). In Trusilk ^®^ and Mersilk ^®^ group, the mean pain VAS score on day 12, 30 and month 3 was recorded as 3.34±6.11 vs. 2.08±1.47, 1.98±0.89 vs. 1.75±0.76, and 1.82±0.85 vs. 1.94±0.94, respectively. On last two visits, i.e., on day 30 and month 3, all the subjects of both arms had no pain, swelling or trismus ( Figure 2a, b and c). Scores of both pain and swelling showed no significant difference between the groups. A decreasing rate of dental bleeding was recorded in subjects of both treatment arms with each follow-up visit ( Figure 4a). Incidence of accidental bleeding or massive bleeding was noted in none of the study participants. Emergency care was not required for any subject after the surgery. A good outcome of surgery was registered for all the subjects. Inferior alveolar nerve paresthesia was reported in one (1.56%) subject randomized to Mersilk ^®^ group during day 7 visit. After the surgery, each subject was followed for signs and symptoms of dry socket. In total, one (1.54%) subject in the Trusilk ^®^ group had dislodged clot on day 7 visit. The subject recovered after treatment, and none of other subjects in both the groups showed further symptoms of dry socket at follow-up visits. Findings of suture loosening and taste change are shown in Table 2. In Mersilk ^®^ group, suture was loosened in one (1.56%) subject on day 3, and in three (4.69%) subjects on day 7 visit. On the other hand, in Trusilk ^®^ group, suture was loosened in four (6.15%) subjects only on day 7 visit. Due to food impaction, a total of three subjects in Trusilk ^®^ group reported to have incidence of wound infection on day 12, day 30 and month 3 visits respectively. Both first and second incidents took place after 8 days of suture removal, while the third incident occurred after 28 days of suture removal. As the suture was removed prior to infection, the incidents were not considered as device-related. The subjects recovered after treatment and reported no further incidence of infection. None of the sutures removed were sent for culture. Time required for wound healing was comparable between the groups ( Table 2). Wound healing scores at three consecutive visits (day 3, 7 and 12) are presented in Figure 4b.

***Adverse events***

During the study period, a total of four (0.78%) mild, non-serious adverse events were noted, and they were not related to the study device. Out of these, two (3.08%) subjects were from Trusilk ^®^ group and two (3.13%) subjects were from Mersilk ^®^ group. In Trusilk ^®^ group, both subjects had upper respiratory tract infection. In Mersilk ^®^ group, one subject had fever and one subject had diarrhea. Any incidence of unexpected serious adverse events, adverse device effect, serious adverse device effect, or unanticipated serious adverse device effect were not recorded during the entire study period.

### Concomitant medication analysis

During the study period, analgesics and antibiotics were prescribed to almost all of the subjects ( Table 4).

**Table 4.** Concomitant or prescribed medications in the study participants during the study period.

Prescribed Medications	Trusilk ^®^ (n=65)	Mersilk ^®^ (n=64)
***Analgesics***		
Diclofenac, n (%)	33 (50.77)	31 (48.44)
Aceclofenac + Serratiopeptidase + Paracetamol, n (%)	19 (29.23)	22 (34.38)
Acetaminophen + Tramadol, n (%)	5 (7.69)	4 (6.25)
***Antibiotics***		
Amoxicillin, n (%)	36 (55.39)	37 (57.81)
Amoxicillin + Clavulanic acid, n (%)	22 (33.85)	23 (35.94)

## Discussion

Impacted third molar removal is essential to minimize the discomfort and complications caused by an unerupted tooth. Pain is one of the most common complications related to impacted third molar tooth. Its prevalence varies between 5 and 53%. On the other hand, the post-operative complication includes incidence of paresthesia, dry socket, infection, hemorrhage along with pain, severity of which depends on the patient’s age, health, and position of the tooth. ^8^ Post-extraction suturing aids in controlling hemorrhage and promoting wound healing. A previous study reported decreased pain, edema, and trismus, and improved wound healing, after using braided silk suture for mucosal closure. ^9^ To our knowledge, no randomized controlled trial has been conducted comparing clinical equivalence of two common silk suture brands for primary mucosa closure after surgical removal of impacted mandibular third molar. The present study compared tissue reaction/inflammation with Trusilk ^®^ and Mersilk ^®^ silk sutures post primary closure of mucosa in subjects undergoing planned surgical removal of impacted mandibular third molar after 3 and 7 days.

Inflammation or swelling, dental caries, oral/dental cysts, and trismus that ultimately result in pain are the usual symptoms associated with impacted mandibular third molars. ^8^ In this study, all the study participants had normal tongue size, and majority of them had full set of teeth (32). Though oral/dental cyst was absent in all the subjects but dental caries, dental scaling and dental deposits, as well as mild swelling and trismus, were recorded in some of the subjects of both study groups. In addition, pre-operative pain was noted in most of the subjects (109/129). Pain, swelling, and trismus, also take place after tooth extraction. ^13^ Incidence of clinical inflammation/tissue reaction such as, pain, swelling and trismus was recorded at all post-operative visits in the subjects of present study. The pain score was highest on the day of the surgery which declined steadily with each passing visit and became nil on the last two follow-up visits. An improvement in rate of swelling and trismus were noted in both treatment arms, Trusilk ^®^ and Mersilk ^®^. Post-operative dental pains are usually not severe and will last for 1-2 days after tooth extraction. ^14^ Likewise, the subjects of the present study received analgesics mostly after the surgery for 72 hours. A marked decrease in prescribed number of analgesics was noted in the next consecutive visits, suggesting a reduction in dental pain.

Silk suture is universally used for dental surgery, as it is easy to handle and also to place knots. ^15^ The intraoperative suture handling parameters were comparable between Trusilk ^®^ and Mersilk ^®^ treatment arms. In both groups, excellent, very good, good and fair scores were recorded, but none of the suture handling characteristics was graded as poor. Mean duration of the surgery was comparable between the groups. At the end of the surgery, good outcome of surgery was marked by both Investigators for all subjects. The rate of paresthesia and dry socket varies from 0.5-20% and 0-35% in patients after removal of impacted teeth. ^8^ Another study on impacted third molar removal complications reported an incidence of 9.2% post-surgical emergency appointments. The majority of them were due to severe pain, swelling and bleeding with a prevalence of 4.8%, 2.6%, and 2.4% respectively. Alveolar osteitis, paresthesia, and trismus with a prevalence of less than 1% were the other reasons for post-surgical emergency appointments. ^16^ Similar to these findings, the present study also recorded incidence of paresthesia in Mersilk ^®^ group (1/64) and dry socket in Trusilk ^®^ group (1/65), 7 days after the tooth extraction. Change in taste was observed in total of two subjects in Trusilk ^®^ group and one subject in Mersilk ^®^ group. However, the changes were temporary, and no further incidence of these complications was noted on the next follow-up visits. Furthermore, post-surgery emergency care was not required for any of the study participants.

Wound infection following impacted third molar extractions is a frequently occurring complication and is considered a risk factor for the healing of the wound. A previous study also reported wound infection in one patient with silk suture. ^17^ The incidence of infection at the site of extraction was observed in three subjects of Trusilk ^®^ group (3/65) on day 12, day 30 and month 3 visits, respectively. As the suture was removed prior to infection, the incidents were not considered as device-related. The suture was loosened in four subjects each of Trusilk ^®^ and Mersilk ^®^ group. However, time required for complete wound healing was comparable between the groups. Additionally, incidence of dental bleeding was observed in majority of subjects of both groups on the day of surgery that declined with each passing visit, reflecting healing in those subjects. The types of adverse events noted in both arms during the study period (4/129) were of low risk, and not related to the suture material.

### Limitations

The limitations of the present study are: (i) potential bias may have occurred in reporting or favoring one suture or another by the staff or surgeons, as they were not blinded, and (ii) all surgical interventions in this trial were clean or clean contaminated elective surgeries, in which risk of infection is minimal and can originate only from contaminants in the operating room environment, or from the surgical team, or oral mucosal colonists present in plaque, cavity or any other soft and hard tissue inflammation.

### Generalizability

The findings of this study can be generalized to the wider population since the study is methodologically robust and appropriately powered to detect a difference in the primary and secondary outcomes. Though the two sutures have been compared in one indication but looking into the clinical equivalence (comparable efficacy and safety) achieved in this study, it’s imperative to state that Trusilk ^®^ suture can be used in all surgeries indicated for Mersilk ^®^ suture.

## Conclusion

Trusilk ^®^ silk suture is clinically equivalent to the Mersilk ^®^ silk suture, as a non-significant difference was observed in clinical inflammation and tissue reaction with respect to swelling, pain and trismus in the extraction area (at 72 hours and 7 days post-surgery and all follow-up visits), incidence of wound infection, and suture loosening, total operative time and amount of anesthesia, intraoperative suture handling parameters, time needed for complete wound healing and suture removal, incidence of other complications such as bleeding and taste changes, and other adverse events among the groups. Both the sutures can be used for mucosal closure after removal of an impacted mandibular third molar with a minimal rate of pain, swelling and trismus.

## Data availability

### Underlying data

Figshare. Trusilk Study Complete PP Data. https://doi.org/10.6084/m9.figshare.20059679.v1. ^18^

This project contains the underlying data related to all the data points mentioned below:

• Demographic data, primary and secondary endpoints

Data are available under the terms of the Creative Commons Attribution 4.0 International license (CC-BY 4.0).

## Reporting guidelines

Figshare: CONSORT check list for ‘Single blind, randomized study comparing clinical equivalence of Trusilk ^®^ and Mersilk ^®^ silk sutures for mucosal closure following surgical removal of mesioangular impacted mandibular third molar’. https://doi.org/10.6084/m9.figshare.20055599.v2. ^19^

Data are available under the terms of the Creative Commons Attribution 4.0 International license (CC-BY 4.0).
