# Supplementary material for: Single blind, randomized study comparing clinical equivalence of Trusilk ® and Mersilk ® silk sutures for mucosal closure following surgical removal of mesioangular impacted mandibular third molar
Source: F1000Res. 2022 Jun 21;11:689. [Version 1] doi: 10.12688/f1000research.122678.1 (PMC9468627; doi:10.12688/f1000research.122678.1)
Supplement: Peer review history

# Peer review: Reviewer response for version 1

**DOI:** 10.5256/f1000research.134700.r145906

**Published:** 2022-09-12

**Authors:** Jingade Krishnojirao Dayashankara Rao

**Affiliations:** 1 Department of Maxillo Facial Surgery & Diagnostic Science, College of Dentistry, Qassim University, Buraida, Saudi Arabia

This is a good clinical work highlighting the benefits of Trusilk ^®^ on comparison to Mersilk®.

 As both sutures are natural non-absorbable black braided sterile silk in nature-Ideally there should not be any significant difference in the outcome of this clinical trial which is in agreement with this study conclusion. The study is well structured and executed.

 Following are some of the minor points which could have been considered to make it more standardised:

1. As per the literature, it is very well known that tissue handling, difficulty of surgery, and duration of surgery play major roles for the development of the study variables which include pain, swelling, and Trismus rather than sutures alone. It might be the sutures per se will play a very minor role here - that too comparing 2 similar sutures.
2. The tissue reaction varies from patient to patient. There is no mention in this study if the comparison is made on the same patients going for bilateral surgical impaction.
3. Double blind study would have been ideal for this study to avoid any operator bias.
4. The Bone cutting technique (burs/tooth section/ combination) should be mentioned so that comparison is standardized. Little info about type of impaction/incision methods should be added as its affects the variables mentioned in this trial.
5. This is multicenter study-which again affects the variables because the tissue reaction is varies among different surgeons for the similar procedure.

The author can clarify the above minor points which would help to strengthen this clinical trial.

Is the work clearly and accurately presented and does it cite the current literature?

Yes

If applicable, is the statistical analysis and its interpretation appropriate?

I cannot comment. A qualified statistician is required.

Are all the source data underlying the results available to ensure full reproducibility?

Yes

Is the study design appropriate and is the work technically sound?

Yes

Are the conclusions drawn adequately supported by the results?

Yes

Are sufficient details of methods and analysis provided to allow replication by others?

Yes

Reviewer Expertise:

Orthognathic surgery, cleft lip & palate, Dental implants, maxillofacial trauma, Minor oral surgeries

I confirm that I have read this submission and believe that I have an appropriate level of expertise to confirm that it is of an acceptable scientific standard.

# Peer review: Reviewer response for version 1

**DOI:** 10.5256/f1000research.134700.r143057

**Published:** 2022-07-25

**Authors:** Abhay Taranath Kamath

**Affiliations:** 1 Department of Oral & Maxillofacial Surgery, KMC Hospital, Mangalore, India

**3rd August 2022: A COI statement was added detailing a collaboration between author and reviewer which was not declared at the time of publishing of this report.**

 This study by Balakrishna *et al.,* is a meticulously planned and systematically executed study as reflected from the methodology of this study. Addressing a common condition (i.e., mesioangular impaction of third molars and the common post operative complication following its removal), the authors have evaluated equivocal influence between use of two suture materials for mucosal closure. While secondary outcome of the intervention are more practically related, the primary outcome like trismus may be not directly correlated to the suture material used instead may be relative to the impaction procedure employed.

 In terms of sample size selection and standardisation of subjects recruited for the study, this study is quite scientific. However the assessment criteria for pain, swelling and trismus used in this study was VAS. VAS is an excellent tool to assess pain subjectively. The validity of assessing swelling using VAS is moderate and its validity in assessing trismus is lower. The authors could have used objective tools routinely used clinical for assessing swelling and degree of trismus. However from use of suture point of view these may not influence its selection, hence I classified it as minor issue.

 The secondary endpoints as addressed in this study are more relevant for clinicians for the selection of type of suture material, these parameters were assessed scientifically.

 The study design does not reflect any major flaws. The result analysis clearly redefines the aim of the study. The authors declare equivalence towards the performance of both the materials. However such an outcome was expected as both the materials do not vary in their composition. Probably by establishing the equivalence the authors intend to allow the clinicians to choose between the providers of this suture material.

 The discussion section of this article describe clinical findings in terms of post operative outcomes with use of both the suture materials. The article describes the limitations and generalizability of the study well.

Is the work clearly and accurately presented and does it cite the current literature?

Yes

If applicable, is the statistical analysis and its interpretation appropriate?

I cannot comment. A qualified statistician is required.

Are all the source data underlying the results available to ensure full reproducibility?

Yes

Is the study design appropriate and is the work technically sound?

Yes

Are the conclusions drawn adequately supported by the results?

Yes

Are sufficient details of methods and analysis provided to allow replication by others?

Yes

Reviewer Expertise:

My areas of expertise and interest are distraction osteogenesis, temporomandibular joint dysfunction and management, maxillofacial trauamatology

I confirm that I have read this submission and believe that I have an appropriate level of expertise to confirm that it is of an acceptable scientific standard.
